# *TNFRSF11B *computational development network construction and analysis between frontal cortex of HIV encephalitis (HIVE) and HIVE-control patients

**DOI:** 10.1186/1476-9255-7-50

**Published:** 2010-09-30

**Authors:** Ju X Huang, L Wang, Ming H Jiang

**Affiliations:** 1 Biomedical Center, School of Electronics Engineering, Beijing University of Posts and Telecommunications, Beijing, 100876, China; 2 Lab of Computational Linguistics, School of Humanities and Social Sciences, Tsinghua Univ., Beijing, 100084, China

## Abstract

**Background:**

*TNFRSF11B *computational development network construction and analysis of frontal cortex of HIV encephalitis (HIVE) is very useful to identify novel markers and potential targets for prognosis and therapy.

**Methods:**

By integration of gene regulatory network infer (GRNInfer) and the database for annotation, visualization and integrated discovery (DAVID) we identified and constructed significant molecule *TNFRSF11B *development network from 12 frontal cortex of HIVE-control patients and 16 HIVE in the same GEO Dataset GDS1726.

**Results:**

Our result verified *TNFRSF11B *developmental process only in the downstream of frontal cortex of HIVE-control patients (*BST2, DGKG, GAS1, PDCD4, TGFBR3, VEZF1 *inhibition), whereas in the upstream of frontal cortex of HIVE (*DGKG, PDCD4 *activation) and downstream (*CFDP1, DGKG, GAS1, PAX6 *activation; *BST2, PDCD4, TGFBR3, VEZF1 *inhibition). Importantly, we datamined that *TNFRSF11B *development cluster of HIVE is involved in T-cell mediated immunity, cell projection organization and cell motion (only in HIVE terms) without apoptosis, plasma membrane and kinase activity (only in HIVE-control patients terms), the condition is vital to inflammation, brain morphology and cognition impairment of HIVE. Our result demonstrated that common terms in both HIVE-control patients and HIVE include developmental process, signal transduction, negative regulation of cell proliferation, RNA-binding, zinc-finger, cell development, positive regulation of biological process and cell differentiation.

**Conclusions:**

We deduced the stronger *TNFRSF11B *development network in HIVE consistent with our number computation. It would be necessary of the stronger *TNFRSF11B *development function to inflammation, brain morphology and cognition of HIVE.

## Background

The neurodegenerative process in HIV encephalitis (HIVE) is associated with cognitive impairment with extensive damage to the dendritic and synaptic structure. Several mechanisms might be involved in including release of neurotoxins, oxidative stress and decreased activity of neurotrophic factors [1]. The effect of HIV on brain has been studied by several researchers. Such as, decreased brain dopamine transporters are related to cognitive deficits in HIV patients with or without cocaine abuse; Magnetic resonance imaging and spectroscopy of the brain in HIV disease; Analysis of the effects of injecting drug use and HIV-1 infection on 18F-FDG PET brain development [2-4]. *TNFRSF11B *computational development network construction and analysis of the frontal cortex of HIV encephalitis (HIVE) is very useful to identify novel markers and potential targets for prognosis and therapy.

*TNFRSF11B *is one out of 50 genes identified as high expression in frontal cortex of HIV encephalitis (HIVE) vs HIVE-control patients. *TNFRSF11B *has been proved to be concerned with molecular function of receptor, and biological process of developmental processes, skeletal development and mesoderm development (DAVID database). *TNFRSF11B*'s relational study also can be seen in these papers [5-10]. However, the molecular mechanism concerning *TNFRSF11B *development construction in HIVE has little been addressed.

In this paper, by integration of gene regulatory network infer (GRNInfer) and the database for annotation, visualization and integrated discovery (DAVID) we identified and constructed significant molecule *TNFRSF11B *development network from 12 frontal cortex of HIVE-control patients and 16 HIVE in the same GEO Dataset GDS1726. Our result verified *TNFRSF11B *developmental process only in the downstream of frontal cortex of HIVE-control patients (*BST2, DGKG, GAS1, PDCD4, TGFBR3, VEZF1 *inhibition), whereas in the upstream of frontal cortex of HIVE (*DGKG, PDCD4 *activation) and downstream (*CFDP1, DGKG, GAS1, PAX6 *activation; *BST2, PDCD4, TGFBR3, VEZF1 *inhibition). Importantly, we datamined that *TNFRSF11B *development cluster of HIVE is involved in T-cell mediated immunity, cell projection organization and cell motion (only in HIVE terms) without apoptosis, plasma membrane and kinase activity (only in HIVE-control patients terms), the condition is vital to inflammation, brain morphology and cognition impairment of HIVE. Our result demonstrated that common terms in both HIVE-control patients and HIVE include developmental process, signal transduction, negative regulation of cell proliferation, RNA-binding, zinc-finger, cell development, positive regulation of biological process and cell differentiation, therefore we deduced the stronger *TNFRSF11B *development network in HIVE consistent with our number computation. It would be necessary of the stronger *TNFRSF11B *development function to inflammation, brain morphology and cognition of HIVE. *TNFRSF11B *development interaction module construction in HIVE can be a new route for studying the pathogenesis of HIVE. Our construction of *TNFRSF11B *development network may be useful to identify novel markers and potential targets for prognosis and therapy of HIVE.

## Methods

### Microarray Data

We used microarrays containing 12558 genes from 12 frontal cortex of HIVE-control patients and 16 HIVE in the same GEO Dataset GDS1726 [1]. HIVE-control patients mean normal adjacent frontal cortex tissues of HIV encephalitis (HIVE) and no extensive damage to the dendritic and synaptic structure.

### Gene Selection Algorithms

50 molecular markers of the frontal cortex of HIVE were identified using significant analysis of microarrays (SAM). SAM is a statistical technique for finding significant genes in a set of microarray experiments. The input to SAM is gene expression measurements from a set of microarray experiments, as well as a response variable from each experiment. The response variable may be a grouping like untreated, treated, and so on. SAM computes a statistic d_i _for each gene i, measuring the strength of the relationship between gene expression and the response variable. It uses repeated permutations of the data to determine if the expression of any genes is significantly related to the response. The cutoff for significance is determined by a tuning parameter delta, chosen by the user based on the false positive rate. We normalized data by log2, and selected two class unpaired and minimum fold change = 1.52. Here we chose the 50 top-fold significant (high expression genes of HIVE compared with HIVE-control patients) genes under the false-discovery rate and q-value as 9.12%. The q-value (invented by John Storey [11]) for each gene is the lowest false discovery rate at which that gene is called significant. It is like the well-known p-value, but adapted to multiple-testing situations.

### Network Establishment of Candidate Genes

The entire network was constructed using GRNInfer [12] and GVedit tools. GRNInfer is a novel mathematic method called GNR (Gene Network Reconstruction tool) based on linear programming and a decomposition procedure for inferring gene networks. The method theoretically ensures the derivation of the most consistent network structure with respect to all of the datasets, thereby not only significantly alleviating the problem of data scarcity but also remarkably improving the reconstruction reliability. The following Equation (1) represents all of the possible networks for the same dataset.

(1) $$J=(X'-A)U\Lambda^{-1}V^{T}+YV^{T}=\hat{J}+YV^{T}$$

We established network based on the 50 top-fold distinguished genes and selected parameters as lambda 0.0 because we used one dataset, threshold 0.000001. Lambda is a positive parameter, which balances the matching and sparsity terms in the objective function. Using different thresholds, we can predict various networks with different edge density.

### Functional Annotation Clustering

The DAVID Gene Functional Clustering Tool provides typical batch annotation and gene-GO term enrichment analysis for highly throughput genes by classifying them into gene groups based on their annotation term co-occurrence [13,14]. The grouping algorithm is based on the hypothesis that similar annotations should have similar gene members. The functional annotation clustering integrates the same techniques of Kappa statistics to measure the degree of the common genes between two annotations, and fuzzy heuristic clustering to classify the groups of similar annotations according to kappa values.

## Results

### Identification of HIVE Molecular Markers

*TNFRSF11B *is one out of 50 genes identified as high expression in frontal cortex of HIV encephalitis (HIVE) vs HIVE-control patients. We normalized data by log2, and selected two class unpaired and minimum fold change = 1.52. Here we chose the 50 top-fold significant (high expression genes of HIVE compared with HIVE-control patients) genes under the false-discovery rate and q-value as 9.12%. We identified potential HIVE molecular markers and chose the 50 top-fold significant positive genes from 12558 genes from 12 frontal cortex of HIVE-control patients and 16 HIVE in the same GEO Dataset GDS1726 including tumor necrosis factor receptor superfamily member 11b (*TNFRSF11B*), programmed cell death 4 (*PDCD4*), diacylglycerol kinase gamma (*DGKG*), craniofacial development protein 1 (*CFDP1*), growth arrest-specific 1 (*GAS1*), paired box 6 (*PAX6*), bone marrow stromal cell antigen 2 (*BST2*), transforming growth factor beta receptor III (*TGFBR3*), vascular endothelial zinc finger 1 (*VEZF1*), etc. (see appendix).

### Identification of *TNFRSF11B *Up- and Down-stream Development Cluster in Frontal Cortex of HIVE-Control Patients and HIVE by DAVID

**Figure 1:**
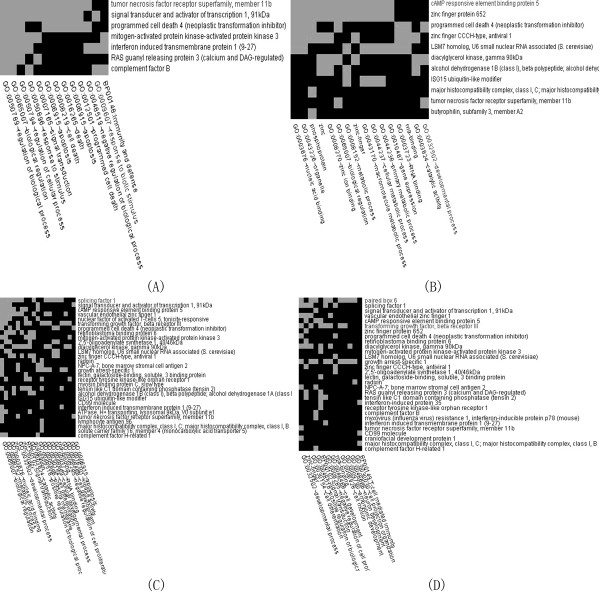
***TNFRSF11B *up- and down-stream development cluster in frontal cortex of HIVE-control patients by DAVID (A, C)**. *TNFRSF11B *up- and down-stream development cluster by DAVID in frontal cortex of HIVE (B, D). Gray color represents gene-term association positively reported, black color represents gene-term association not reported yet.

We first datamined 4 lists of *TNFRSF11B *up- and down-stream genes from 12 frontal cortex of HIVE-control patients and 16 HIVE by GRNInfer respectively. With inputting 4 lists into DAVID, we further identified *TNFRSF11B *up- and down-stream development cluster of HIVE-control patients and HIVE. *TNFRSF11B *development cluster terms only in frontal cortex of HIVE-control patients cover apoptosis, plasma membrane and kinase activity, as shown in (Figure 1A, C). However, *TNFRSF11B *development cluster terms only in frontal cortex of HIVE contain T-cell mediated immunity, cell projection organization and cell motion, as shown in (Figure 1B, D). *TNFRSF11B *development cluster terms both in frontal cortex of HIVE-control patients and HIVE include developmental process, signal transduction, negative regulation of cell proliferation, RNA-binding, zinc-finger, cell development, positive regulation of biological process and cell differentiation, as shown in (Figure 1A, B, C, D).

In frontal cortex of HIVE-control patients, *TNFRSF11B *upstream showed little results without developmental process, as shown in (Figure 1A). In frontal cortex of HIVE, *TNFRSF11B *upstream modules mainly cover developmental process (*DGKG, PDCD4, TNFRSF11B*), etc., as shown in (Figure 1B). In frontal cortex of HIVE-control patients, *TNFRSF11B *downstream modules mainly consist of developmental process (*BST2, DGKG, GAS1, PDCD4, TGFBR3, VEZF1, TNFRSF11B*), etc., as shown in (Figure 1C). In frontal cortex of HIVE, *TNFRSF11B *downstream modules mainly contain developmental process (*CFDP1, DGKG, BST2, PDCD4, GAS1, PAX6, TGFBR3, VEZF1, TNFRSF11B*), etc., as shown in (Figure 1D).

### *TNFRSF11B *Up- and Down-stream Development Network Construction in Frontal Cortex of HIVE-Control Patients and HIVE

**Figure 2:**
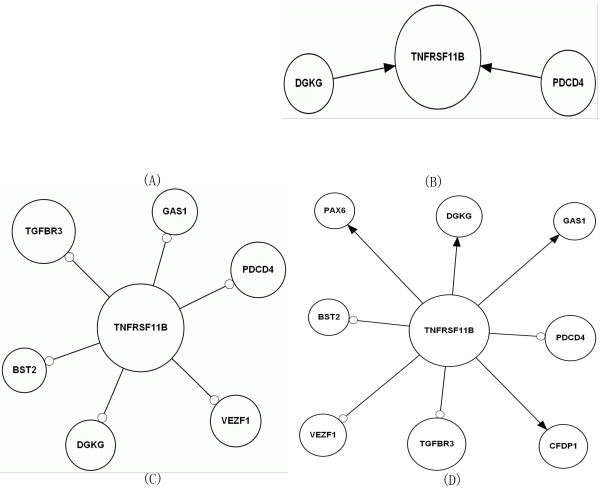
***TNFRSF11B *up- and down-stream development network construction in frontal cortex of HIVE-control patients by infer (A, C)**. *TNFRSF11B *up- and down-stream development network construction in frontal cortex of HIVE by infer (B, D). Arrowhead represents activation, empty cycle represents inhibition.

In frontal cortex of HIVE-control patients, *TNFRSF11B *upstream development network appeared no result, as shown in (Figure 2A), whereas in frontal cortex of HIVE, *TNFRSF11B *upstream development network showed that *DGKG, PDCD4 *activate *TNFRSF11B*, as shown in (Figure 2B).

In frontal cortex of HIVE-control patients, *TNFRSF11B *downstream development network reflected that *TNFRSF11B *inhibits *BST2, DGKG, GAS1, PDCD4, TGFBR3, VEZF1*, as shown in (Figure 2C), whereas in frontal cortex of HIVE, *TNFRSF11B *downstream development network appeared that *TNFRSF11B *activates *CFDP1, DGKG, GAS1, PAX6 *and inhibits *BST2, PDCD4, TGFBR3, VEZF1*, as shown in (Figure 2D).

## Discussion

We have already done some works in this relative field about gene network construction and analysis presented in our published papers [15-19]. By integration of gene regulatory network infer (GRNInfer) and the database for annotation, visualization and integrated discovery (DAVID) we constructed significant molecule *TNFRSF11B *development network and compared *TNFRSF11B *up- and down-stream gene numbers of activation and inhibition between HIVE-control patients and HIVE (Table 1).

**Table 1:** Up- and down-stream gene numbers of activation and inhibition of each module with *TNFRSF11B *gene in *TNFRSF11B *development cluster between frontal cortex of HIVE-control patients and HIVE.

Term	*TNFRSF11B *upstream				*TNFRSF11B *downstream			
	
	con(act)	con(inh)	exp(act)	exp(inh)	con(act)	con(inh)	exp(act)	exp(inh)
Apoptosis	1	1			1	1		

Signal Transduction	2	1			4	4	4	5

Developmental Process			2	0	0	6	4	4

con represents control (HIVE-control patients), exp: experiment (HIVE), act: activation, inh: inhibition.

In *TNFRSF11B *developmental process of upstream network of frontal cortex of HIVE-control patients there was no result, whereas in that of HIVE, our integrative result reflected that *DGKG, PDCD4 *activate *TNFRSF11B*. In *TNFRSF11B *developmental process of downstream network of HIVE-control patients, our integrative result illustrated that *TNFRSF11B *inhibits *BST2, DGKG, GAS1, PDCD4, TGFBR3, VEZF1*, whereas in that of HIVE, *TNFRSF11B *activates *CFDP1, DGKG, GAS1, PAX6 *and inhibits *BST2, PDCD4, TGFBR3, VEZF1 *(Figure 1, 2; Table 2). *PAX6 *is identified by molecular function of transcription factor, homeobox transcription factor, nucleic acid binding and DNA-binding protein, and it is involved in biological process of nucleoside, nucleotide and nucleic acid metabolism, mRNA transcription, mRNA transcription regulation, developmental processes, neurogenesis, segment specification and ectoderm development (DAVID database). *PAX6*'s relational study also can be presented in these papers [20-25]. *DGKG *has been proved to be concerned with molecular function of kinase, and biological process of lipid, fatty acid and steroid metabolism, signal transduction, intracellular signaling cascade and lipid metabolism (DAVID). *DGKG*'s relational study also can be presented in these papers [26-29]. *GAS1*'s molecular function consists of mRNA processing factor, mRNA splicing factor, kinase modulator, dehydrogenase and kinase activator, and it is concerned with biological process of glycolysis, amino acid catabolism, pre-mRNA processing, mRNA splicing, cell proliferation and differentiation (DAVID database). *GAS1*'s relational study also can be presented in these papers [30-33]. *PDCD4 *is relevant to molecular function of nucleic acid binding, translation factor, translation elongation factor and miscellaneous function, and biological process of protein metabolism and modification, protein biosynthesis, apoptosis, induction of apoptosis (DAVID). *PDCD4*'s relational study also can be presented in these papers [34-39]. *CFDP1 *has been reported to have molecular function of mRNA splicing factor, select calcium binding proteins and KRAB box transcription factor, and to be concerned with biological process of mRNA transcription regulation and cell motility (DAVID database). *CFDP1*'s relational study also can be presented in these papers [40-44]. We gained the positive result of *TNFRSF11B *developmental process through the net numbers of activation minus inhibition compared with HIVE-control patients and predicted possibly the increase of *TNFRSF11B *developmental process in HIVE.

**Table 2:** Activation and inhibition gene names of *TNFRSF11B *up- and down-stream development cluster in frontal cortex of HIVE-control patients and HIVE.

Term	*TNFRSF11B *upstream			
	
	con(act)	con(inh)	exp(act)	exp(inh)
Developmental process			*DGKG, PDCD4*	

**Term**	***TNFRSF11B *downstream**			
	
	**con(act)**	**con(inh)**	**exp(act)**	**exp(inh)**

Developmental process		*BST2, DGKG, GAS1, PDCD4, TGFBR3, VEZF1*	*CFDP1, DGKG, GAS1, PAX6*	*BST2, PDCD4, TGFBR3, VEZF1*

con represents control (HIVE-control patients), exp: experiment (HIVE), act: activation, inh: inhibition.

Importantly, we datamined that *TNFRSF11B *development cluster of HIVE is involved in T-cell mediated immunity, cell projection organization and cell motion (only in HIVE terms) without apoptosis, plasma membrane and kinase activity (only in HIVE-control patients terms), the condition is vital to inflammation, brain morphology and cognition impairment of HIVE. Our result demonstrated that common terms in both HIVE-control patients and HIVE include developmental process, signal transduction, negative regulation of cell proliferation, RNA-binding, zinc-finger, cell development, positive regulation of biological process and cell differentiation, therefore we deduced the stronger *TNFRSF11B *development network in HIVE consistent with our number computation. Some researchers indicated that tumor necrosis factor receptor studied to relate with inflammation, brain morphology and cognition [45,46]. Therefore, we predicted the stronger *TNFRSF11B *development function in HIVE. It would be necessary of the stronger *TNFRSF11B *development function to inflammation, brain morphology and cognition of HIVE.

## Conclusions

In summary, we deduced the stronger *TNFRSF11B *developmental process in HIVE. It would be necessary of the stronger *TNFRSF11B *development function to inflammation, brain morphology and cognition of HIVE. *TNFRSF11B *development interaction module construction in HIVE can be a new route for studying the pathogenesis of HIVE.

## Abbreviations

TNFRSF11B: tumor necrosis factor receptor superfamily member 11b; IFI44L: interferon-induced protein 44-like; ADH1B: alcohol dehydrogenase 1B (class I) beta polypeptide; RASGRP3: RAS guanyl releasing protein 3; MAPKAPK3: mitogen-activated protein kinase-activated protein kinase 3; CREB5: cAMP responsive element binding protein 5; MX1: myxovirus resistance 1 interferon-inducible protein p78; IFITM1: interferon induced transmembrane protein 1; MYBPC1: myosin binding protein C slow type; ROR1: receptor tyrosine kinase-like orphan receptor 1; IFI35: interferon-induced protein 35; LCAT: lecithin-cholesterol acyltransferase; ZC3HAV1: zinc finger CCCH-type antiviral 1; LY96: lymphocyte antigen 96; TSPAN4: tetraspanin 4; C10orf116: chromosome 10 open reading frame 116; DGKG: diacylglycerol kinase gamma; STAT1: signal transducer and activator of transcription 1; IFI27: interferon alpha-inducible protein 27; BST2: bone marrow stromal cell antigen 2; TGFBR3: transforming growth factor, beta receptor III; SLC16A4: solute carrier family 16 member 4; FER1L3: myoferlin; ZNF652: zinc finger protein 652; HLA-B: hypothetical protein LOC441528; PDCD4: programmed cell death 4; SF1: splicing factor 1; CFHR1: complement factor H-related 1; CFB: complement factor B; LGALS3BP: lectin galactoside-binding soluble 3 binding protein; RDX: radixin; MT1G: metallothionein 1G; RBBP6: retinoblastoma binding protein 6; TENC1: tensin like C1 domain containing phosphatase; PAX6: paired box 6; NFAT5: nuclear factor of activated T-cells 5 tonicity-responsive; DGKG: diacylglycerol kinase, gamma; CFDP1: craniofacial development protein 1; VEZF1: vascular endothelial zinc finger 1; GAS1: growth arrest-specific 1; ATP6V0E1: ATPase H+ transporting lysosomal 9 kDa V0 subunit e1.

## Competing interests

The authors declare that they have no competing interests.

## Authors' contributions

All authors participated in design and performance of the study, interpreted the result and contributed to writing the paper. All authors read and approved the final version of the manuscript.
